# Social consequences of COVID-19 on fertility preference consistency and contraceptive use among Nigerian women: insights from population-based data

**DOI:** 10.1186/s40834-022-00181-0

**Published:** 2022-08-02

**Authors:** Joshua O. Akinyemi, Oluwafemi I. Dipeolu, Ayodeji M. Adebayo, Babatunde M. Gbadebo, Grace A. Ajuwon, Tubosun A. Olowolafe, Yemi Adewoyin, Clifford O. Odimegwu

**Affiliations:** 1 Department of Epidemiology and Medical Statistics, Faculty of Public Health, College of Medicine, University of Ibadan, Ibadan, Nigeria; 2 Infectious Diseases Institute, College of Medicine, University of Ibadan, Ibadan, Nigeria; 3 Demography and Population Studies Programme, Schools of Public Health and Social Sciences, University of the Witwatersrand, Johannesburg, South Africa; 4 Department of Health Promotion and Education, Faculty of Public Health, College of Medicine, University of Ibadan, Ibadan, Nigeria; 5 Department of Community Medicine, Faculty of Public Health, College of Medicine, University of Ibadan, Ibadan, Nigeria; 6 E.Latunde Odeku Medical Library, College of Medicine, University of Ibadan, Ibadan, Nigeria; 7 Department of Geography, University of Nigeria, Nsukka, Nigeria

**Keywords:** COVID-19 lockdown, Fertility preference, Childbearing intention, Modern contraceptive use, Nigeria

## Abstract

**Background:**

Emerging evidence from high income countries showed that the COVID-19 pandemic has had negative effects on population and reproductive health behaviour. This study provides a sub-Saharan Africa perspective by documenting the social consequences of COVID-19 and its relationship to fertility preference stability and modern contraceptive use in Nigeria.

**Method:**

We analysed panel data collected by Performance Monitoring for Action in Nigeria. Baseline and Follow-up surveys were conducted before the COVID-19 outbreak (November 2019-February 2020) and during the lockdown respectively (May-July 2020). Analysis was restricted to married non-pregnant women during follow-up (*n* = 774). Descriptive statistics and generalized linear models were employed to explore the relationship between selected social consequences of COVID-19 and fertility preferences stability (between baseline and follow-up) as well as modern contraceptives use.

**Results:**

Reported social consequences of the pandemic lockdown include total loss of household income (31.3%), food insecurity (16.5%), and greater economic reliance on partner (43.0%). Sixty-eight women (8.8%) changed their minds about pregnancy and this was associated with age groups, higher wealth quintile (AOR = 0.38, CI: 0.15-0.97) and household food insecurity (AOR = 2.72, CI: 1.23-5.99). Fertility preference was inconsistent among 26.1%. Women aged 30-34 years (AOR = 4.46, CI:1.29-15.39) were more likely of inconsistent fertility preference compared to 15-24 years. The likelihood was also higher among women with three children compared to those with only one child (AOR = 3.88, CI: 1.36-11.08). During follow-up survey, 59.4% reported they would feel unhappy if pregnant. This was more common among women with tertiary education (AOR = 2.99, CI: 1.41-6.33). The odds increased with parity. The prevalence of modern contraceptive use was 32.8%. Women aged 45-49 years (AOR = 0.24, CI: 0.10-0.56) were less likely to use modern contraceptives than those aged 15-24 years. In contrast, the odds of contraceptive use were significantly higher among those with three (AOR = 1.82, CI: 1.03-3.20), four (AOR = 2.45, CI: 1.36-4.39) and at least five (AOR = 2.89, CI: 1.25-6.74) children. Unhappy disposition towards pregnancy (AOR = 2.48, CI: 1.724-3.58) was also a significant predictor of modern contraceptive use.

**Conclusion:**

Some social consequences of COVID-19 affected pregnancy intention and stability of fertility preference but showed no independent association with modern contraceptive use.

## Background

The outbreak of the COVID-19 pandemic has had diverse consequences on livelihood and health across the globe. Earlier in the outbreak, lockdown, physical distancing, and other public health measures to control the spread caused disruptions to healthcare services and economic activities [1–3]. The direct and indirect effects of the pandemic and its control measures vary from one context to another. For instance, while the direct effect such as the number of cases and fatalities has been overwhelming in many developed countries but fewer in sub-Saharan Africa (SSA), the indirect effects have been sources of concern in most of SSA [4]. The fragile health system and weak economic base in SSA exacerbated the hardship occasioned by lockdown and other containment measures [5]. These multiplier effects have been reported to have negative outlooks for health indices such as sexual and reproductive health (SRH), maternal and child health (MCH), as well as HIV care and treatment services [6]. These are areas where efforts and investment by several stakeholders and development partners have yielded some progress for SSA in the last decade [7].

It’s been argued that the fertility trajectories in the post-pandemic era would be driven by effects of the various social measures aimed at its control. And these would vary across developmental context, stage of demographic transition, age structure and availability of modern contraceptives and reproductive technologies [8, 9]. For low- and middle-income countries with poor economic development and limited access to contraceptives, fertility is likely to increase. Emerging evidence on the short-term effects of the COVID-19 pandemic on sexual and reproductive health (SRH) confirmed some of the projections about service disruptions and its sequalae. For instance, access to family planning services and abortion care were affected in some SSA countries [2, 3, 6]. These could translate into increased unintended pregnancies, unsafe abortions, maternal morbidity, and mortality. Many of the latter issues would require more time for empirical data to accrue.

On a positive note, evidence about access to contraceptives and its usage in SSA at the peak of the first COVID-19 lockdown suggests that the problems may not be as grievous as earlier envisaged. For instance, longitudinal data from Kenya and Burkina Faso showed that over a six-month period, there was no change in use of contraceptives among women at risk of unintended pregnancy and the proportion of women who opted for a more effective method was higher [10]. Besides, very few non-contracepting women implicated COVID-19 as reasons for non-use. Furthermore, a similar study that included data from Nigeria and Burkina Faso revealed that the need for and use of contraceptives increased, especially in rural areas of Kenya and Burkina Faso [11]. There were differences across contexts and socio-economic characteristics, which are not unexpected based on past knowledge about the roles of these background variables [10, 11].

Aside from the foregoing, the fertility or reproductive intentions that drive contraceptive behaviour are also important and deserve empirical assessment in light of COVID-19 lockdown and its aftermaths. Before the pandemic, the literature indicates that women fertility intentions often change over time in response to prevailing economic situations and other considerations such as employment and marital contexts [12–14]. In the pandemic era, studies conducted in Australia [15], United Kingdom [16], the United States [17], Italy [18], Poland [19] and Shanghai, China [20] revealed that on the average, about one-third of women or couples who planned pregnancy for Jan-July 2020 postponed their pregnancy intention mostly due to concerns about income loss, access to MCH services and possible effects of COVID-19 on pregnancy. Fertility behaviours in these high-income countries differ from SSA countries, especially those with high fertility.

It is widely known that sub-regions of SSA are in different stages of fertility transition [21]. Countries in Southern and Eastern Africa have mostly transited to low fertility regime, while Western and Central Africa have remained persistently high fertility settings with very slow transitions [21, 22]. Therefore, fertility intention and the response to COVID-19 lockdown restrictions may also differ. The pre-pandemic literature on fertility preferences and intentions indicate that there is some level of instability and depend on factors such as household economic status, education, and parity [12, 14, 22]. Assessment of fertility intention and/or preferences provides the necessary benchmark for family planning advocacy and gives insight into future fertility trajectory for a population [13]. In addition, fertility intention measures are employed for estimation of family planning needs and demand satisfaction which are important indicators of SDG3 target on family planning uptake.

This study aims to provide evidence on childbearing intentions amidst the COVID-19 pandemic in Nigeria, the most populous country in West Africa. A previous study in the country showed that the proportion of women who wanted more children declined between 2003 and 2013, but this was more likely in the South West region, educated women and urban dwellers. In contrast, the percentage undecided about future childbirth was higher in 2013 compared to 2003 and was associated with parity and discrepancies in desired family sizes among couples [23]. We hypothesise that COVID-19 and its attendant adverse economic effects may heighten the level of uncertainty. Therefore, reproductive-age women may have inconsistent childbearing desires, especially during the lockdown restrictions. To explore these issues, our study aimed to address the following objectives: (i) assess the social consequences of COVID-19 among married women in Nigeria; (ii) investigate short-term changes in childbearing intentions due to COVID-19 concerns; (iii) explore the relationship between social consequences of COVID-19 and stability of fertility preference, and (iv) investigate the implication of social consequences of COVID-19 and stability of fertility preference on modern contraceptive use among married women in Nigeria. Our results provide contextual evidence on the short-term effects of COVID-19 lockdown on fertility intentions among Nigerian women. It can also serve as a benchmark for future studies on the medium and long-term impact of the pandemic on reproductive behaviours.

## Methods

### Study setting

In this study, we analysed data for Lagos and Kano States, the two most populous States in Nigeria, with 2016 projected population of 12.6 million and 13.1 million, respectively [24]. The first case of COVID-19 in Nigeria was detected in late February 2020, with the number of cases rising to 195,890 as of 6th September 2021 and a case fatality of 1.3%. Being the commercial capital, Lagos is the epicentre of the pandemic in Nigeria, with 74,044 cases and 648 deaths as of 6 September 2021. Kano, the commercial hub in the North, has recorded 4102 cases with 111 deaths. Total lockdown and restriction of inter-state travels were implemented in the entire country between May and July 2020.

The reproductive health indices in Lagos and Kano States contrast with each other. For instance, while the total fertility rate in Lagos was 3.4, Kano had 6.5. Estimates of modern contraceptive prevalence rate in Lagos and Kano was 29.0 and 5.6%, respectively [25]. Underlying these differences are variations in educational attainment and other socio-economic variables.

### Data source

This study sourced data from Performance Monitoring for Action (PMA) which is a panel survey implemented in nine countries across Sub-Saharan Africa and Southeast Asia. PMA collects representative population-based data to monitor trends in selected reproductive health and family planning indicators. Detailed methodology and summary findings from PMA are available online and in previous publications that used the data [10, 11].

PMA surveys have been implemented in Nigeria since 2017, with four rounds of data collection successfully conducted. In this study, we analysed data from two rounds. These were the panel baseline survey conducted in December 2019- January 2020 and the follow-up survey which was implemented in May-July 2020 to track reproductive indices in response to the COVID-19 outbreak. Detailed descriptions of the sampling process and data collection for the baseline and COVID-19 follow-up surveys in Nigeria are available online [26]. In brief, the PMA surveys in Nigeria took place in Lagos and Kano States, where representative data were collected on knowledge and practice of contraceptive use in 52 and 25 enumeration areas (EAs), respectively. The EAs and females aged 15-49 years were selected via a multi-stage stratified cluster design. Data were collected by trained fieldworkers using face-face interviews.

The follow-up data collection coincides with the peak of lockdown and other measures introduced to contain further spread of the pandemic in Nigeria. During the follow-up survey, telephone interviews were conducted among baseline respondents who gave consent to be recontacted and had access to a phone. In Lagos and Kano, the follow-up sample was 82.6 and 33.6% of the baseline population, respectively.

### Analytic sample

Out of the 1603 (Lagos:1174 and Kano:429) respondents eligible for the follow-up survey, 1346 were successfully interviewed. Of this number, we analysed data on a weighted sample of 774 women who were married [married or cohabiting with a man] and not pregnant during the follow-up survey. The follow-up sample was reverse weighted to properly account for phone ownership, consent and completeness of follow-up interview [11, 26]. Pregnant women were excluded from analyses because they did not respond to the questions that constituted our outcome measures in this study.

### Outcome measures

We analysed four outcome measures described as follows:

i. Change in pregnancy intention due to COVID-19 concerns. This was analysed as a dichotomous variable coded as Yes (1) or No (0). The variable was derived from the response to the question, “*Have you changed your mind about wanting to get pregnant due to concerns about Coronavirus (COVID-19)?*” This data was collected during the follow-up survey.
ii. Inconsistent fertility preference. This was an indicator variable derived from data collected at baseline and during follow-up. The question posed to respondents was, “*Now I have some questions about the future. Would you like to have a (another) child, or would you prefer not to have any (more) children?*”. The response options were: “Have a/another child”; “No more/prefer no children”; “Can’t get pregnant”; “Undecided/ Don’t know”. Any respondent whose response in the follow-up survey differ from their answer at baseline were scategorised as having “inconsistent fertility preference (1)”, while those whose responses remained the same between baseline and follow-up were classified as consistent (0).
iii. Disposition to pregnancy: During the follow-up survey, respondents were asked, “*If you got pregnant now, how would you feel?*”. The response categories were: “very happy”, “happy”, “mixed happy and unhappy”, “sort of unhappy”, “very unhappy”. The first two options were recoded as happy (0), while the other three options were recoded as unhappy (1).
iv. Modern contraceptive use: this was a dichotomous variable coded as Yes [1] or no [0] for users and non-users of modern family planning methods respectively.

### Explanatory variables

Three categories of explanatory variables were analysed. First are the social consequences of COVID-19 restrictions, which constitute the main independent variables in this study. These were all based on the following data collected in the follow-up survey:

(i) Loss of personal income: this was based on the question, “*Since Coronavirus restrictions began, how much of a loss of income have you experienced*”. The options were Large, Moderate, Small, and No income.
(ii) Loss of household income: this was based on the question, “*Since Coronavirus restrictions began, how much of a loss of income has your household experienced*”. The response options were None, Complete, Partial. In the data, no respondent selected “none”. Therefore, only two categories were reported in the analysis.
(iii) Worry about future household finance: this was elicited from response to the question, “*Are you worried about the impact of Coronavirus (COVID-19) on your household’s finances in the future?*” The response was either Yes or No.
(iv) Household food insecurity: Participants were rated as having household food insecurity if they answered in the affirmative to the question, “*Since Coronavirus restrictions began, did you or any household member go a whole day and night without eating anything because there was not enough food?*”
(v) Economic reliance on the partner: This was an indicator variable coded as Yes or No. The base question was, “*Are you more economically reliant on your husband/partner now than before the Coronavirus restrictions began*?” Respondents who answered “Yes” were classified as being economically reliant on the partner.

The second group of explanatory variables comprises socio-demographic characteristics of participants, and this include age, highest educational attainment, employment, wealth quintile, and type of residence. Age was classified as 15-24, 25-29, 30-34, 35-39, 40-44, 45-49 years. The categories for education include none, primary, secondary, and higher, while employment was a dichotomous variable coded as Yes or No. The type of residence was either rural or urban. Wealth quintile was derived from the application of principal component analysis to various household items possessed by respondents. The first component from PCA was subsequently ranked and divided into 5 categories lowest, lower, middle, higher, and highest. The relationship between these socio-demographic variables and fertility intentions or behaviour is well illustrated in the literature. For instance, there is an inverse relationship between age and fertility desire, such that as age increases, childbearing desire reduces [27]. A similar inverse relationship exists between educational attainment, employment, wealth quintile and fertility [23, 28, 29].

The third set of explanatory variables were the marital and reproductive profile of the women. These include the number of times married/cohabited, family type, children-ever-born and history of pregnancy termination in the past 3 years. The number of times married was classified as either one or more than one. Evidence from the literature suggest that women who have been married more than once tend to be pronatal [30, 31]. The family type was either monogamy or polygyny (partner has other wives). A positive relationship exists between polygyny and fertility intentions [32]. Children-ever-born (CEB) was classified as < 1, 2, 3, 4, and 5+. It is expected that fertility desires reduce with CEB [23].

### Statistical analysis

The first stage of data analysis involved a description of participants characteristics. Frequencies and percentages were generated for all the outcome measures and the explanatory variables. Thereafter, each outcome measure was analysed to investigate associations with the explanatory variables. A complementary log-log model was employed for change in pregnancy intention because it was a rare outcome [33]. Less than 10% of respondents reportedly changed their minds about pregnancy due to COVID-19 concerns. For the other three outcomes (disposition to pregnancy, consistency of childbearing desire and modern contraceptive use], a binomial model with a logit link function was implemented [34]. The complementary log-log model was of the form:

$$\Pr \left({y}_i=1|{x}_i\right)=1-\exp \left[-\exp \left({\gamma}_1{A}_i+{\gamma}_2{B}_i+{\varepsilon}_i\right)\right]$$

Where *y*_*i*_ = Change in pregnancy intention by respondent *i.*

The binomial model implemented was:

$${y}_i={\gamma}_1{A}_i+{\gamma}_2{B}_i+{\varepsilon}_i$$

Where

$${y}_i=\mathit{\log}\left(\frac{p_i}{1-{p}_i}\right)$$

*p*_*i*_ is the probability of the outcome measure [unstable childbearing desire, or unhappy disposition to pregnancy or modern contraceptive use] for *i*^*th*^ respondent. *γ*_1_ and *γ*_2_ represent the coefficients for social consequences of COVID-19 restrictions and other explanatory variables, respectively. *A*_*i*_ and *B*_*i*_ were social consequences of COVID-19 restrictions and other background variables included in the models, respectively. *ε*_*i*_ is the error term which was assumed to follow the binomial distribution.

For all outcome measures, multivariable models were built in stages. The first stage was a univariate model with one variable at a time. From these, the Unadjusted Odds Ratio (OR) were estimated. At the second stage, all the measures of social consequences of COVID-19 were entered into the model. In the last stage, variables with *p*-values less than 0.1 from the univariate and stage two models were entered to determine the independent relationship between the social consequences measure and the outcome variable. We employed robust standard error with proper control for the sampling weight for the follow-up surveys for all models. This helped to account for the clustering of participants selected from the same enumeration area (primary sampling unit).

### Ethical considerations

The protocol for the PMA panel surveys was approved by the Lagos State University Teaching Hospital Health Research Ethics Committee. Respondents provided written informed consent before participation in the interviews. We obtained approval from PMA for data retrieval and analysis. The final data analysed did not contain any identifying information.

## Results

### Characteristics of study participants

The mean age of participants was 35.2 (SD = 7.5) years (Table 1). Age groups 30-34 (23.4%) and 35-39 (23.3%) had the highest number of respondents. One hundred and three (13.4%) had no formal education while higher proportions attained secondary (39.2%) and tertiary education (30.5%). The majority of respondents (82.8%) were employed and reside in the urban areas (81.8%).

**Table 1:** Characteristics of study participants

Variables (***n*** = 774)	Freq	Percentage (%)
**Socio-demographics**		
Age (Years): Mean (SD)	35.19 (7.52)	
*Age group (Years)*		
15-24	76	9.8
25-29	130	16.8
30-34	181	23.4
35-39	180	23.3
40-44	136	17.6
45-49	71	9.1
*Highest education*		
None	103	13.4
Primary	131	16.9
Secondary	304	39.2
Tertiary	236	30.5
*Employment*		
Yes	640	82.8
No	134	17.3
*Place of residence*		
Urban	633	81.8
Rural	141	18.2
*Wealth quintile*		
Lowest	124	16.1
Lower	136	17.6
Middle	154	19.9
Higher	180	23.3
Highest	180	23.3
*State*		
Lagos	548	70.9
Kano	226	29.1
**Social consequences of COVID-19 restrictions**		
*Loss of personal income*		
Small	77	9.9
Moderate	208	26.9
Large	192	24.9
No income	297	38.3
*Loss of household income*		
Some or partial	532	68.7
Total	242	31.3
*Household food insecurity*		
No	646	83.5
Yes	128	16.5
*Worry about future household finance*		
No	60	7.6
Yes	714	92.4
*Economic reliance on partner*		
No	441	57.0
Yes	333	43.0
**Marital and reproductive characteristics**		
*No of times married/cohabit*		
Once	699	90.3
More than one	75	9.7
*Family type*		
Monogamy	611	78.9
Polygynous	163	21.1
*Children ever-born*		
< =1	139	18
2	163	21
3	160	20.6
4	144	18.6
> =5	168	21.8
*History of pregnancy termination in past 3 years*		
No	704	91.0
Yes	70	9.0

The marital profile of study participants showed that most of them (90.3%) have been married/cohabited only once in their lifetime (Table 1). In terms of family type, 21.1% were in a polygynous relationship. Their reproductive history showed an even distribution such that about one-fifth has had at most one child, two, three, four and five or more children. Only 9.0% reported a history of pregnancy termination within 3 years before the survey.

### Social consequences of COVID-19 restrictions

About half of the participants reported a moderate to large loss of personal income due to COVID-19 restrictions (Table 1). Similarly, 31.3% have experienced a total loss of household income. Household food insecurity was reported by 16.5%, while the majority (92.4%) were also worried about future household finance. About four out of 10 women (43.0%) had become more economically reliant on their partners since the outbreak of COVID-19.

### Changes in pregnancy intention due to COVID-19 concerns

Sixty-eight (8.8%) of the 774 women had changed their minds about getting pregnant due to COVID-19 concerns. The proportion across different background characteristics is presented in the second column of Table 2. The percentage who had changed mind about pregnancy declined from 22.7% in age 15-24 years to 5.9% among those aged 45-49 years. There were no major differentials in the percentage across education, employment, and residence. However, the proportion also varied from 17.2% in the lowest wealth quintile to 7.4% in the highest quintile.

**Table 2:** Frequency and factors associated with change in pregnancy intention due to COVID-19 concerns

Variables	Change in pregnancy intention due to COVID-19 concerns			
Socio-demographics	(%)	Unadjusted OR (95% CI)	Adjusted OR (95% CI)	
*Age group (Years)*			**Model I**	**Model II**
15-24	22.7	ref		ref
25-29	9.4	0.38(0.15-0.99)*		0.41(0.17-0.98)*
30-34	8.5	0.34(0.14-0.85)*		0.37(0.14-0.96)*
35-39	6.9	0.28(0.12-0.67)*		0.32(0.15-0.68)*
40-44	5.1	0.20(0.07-0.58)*		0.25(0.09-0.69)*
45-49	5.9	0.23(0.07-0.80)*		0.33(0.09-1.15)
*Highest education*				
None	5.8	ref		
Primary	14.1	2.52(0.58-10.90)		
Secondary	9.3	1.62(0.47-5.66)		
Tertiary	6.6	1.14(0.32-4.02)		
*Employment*				
Yes	7.9	ref		
No	13.2	1.72(0.76-3.93)		
*Place of residence*				
Urban	8.7	ref		
Rural	9.6	1.12(0.39-3.23)		
*Wealth quintile*				
Lowest	17.2	ref		ref
Lower	6.2	0.34(0.13-0.90)*		0.32(0.14-0.76)*
Middle	8.5	0.47(0.20-1.13)		0.46(0.19-1.12)
Higher	6.8	0.37(0.11-1.26)		0.38(0.15-0.97)*
Highest	7.4	0.41(0.18-0.93)*		0.50(0.24-1.04)
*State*				
Lagos	7.3	ref		
Kano	12.6	1.79(0.78-4.09)		
**Social consequences of COVID-19**				
*Loss of personal income*				
Small	3.2	ref	ref	
Moderate	5.6	1.78(0.30-10.53)	1.83(0.30-11.13)	
Large	11.4	3.76(0.72-19.61)	3.54(0.67-18.57)	
No income	11.0	3.61(0.70-18.71)	0.14(0.01-1.48)	
*Loss of household income*				
Some or partial	6.8	ref	ref	ref
Total	13.3	2.04(0.98-4.27)	25.64(3.07-214.43)	1.64(0.86-3.13)
*Household food insecurity*				
No	7.0	ref	ref	ref
Yes	18.3	2.79(1.29-6.04)*	2.34(1.07-5.12)	2.72(1.23-5.99)*
*Worry about future household finance*				
No	4.4	ref	ref	
Yes	9.2	2.14(0.64-7.11)	1.43(0.42-4.90)	
*Economic reliance on partner*				
No	6.2	ref	ref	ref
Yes	12.3	2.04(1.01-4.12)*	2.08(1.04-4.16)	1.80(0.82-3.96)
**Marital and reproductive characteristics**				
*No of times married/cohabitted*				
Once	8.7	ref		
More than one	10.3	1.20(0.44-3.27)		
*Family type*				
Monogamy	9.3	ref		
Polygynous	7.3	0.78(0.34-1.76)		
*Children ever-born*				
< =1	11.1	ref		
2	6.5	0.57(0.21-1.57)		
3	10	0.90(0.32-2.53)		
4	10.8	0.97(0.35-2.74)		
> =5	6.4	0.56(0.18-1.70)		
*History of pregnancy termination in past 3 years*				
No	9.1	ref		
Yes	6.4	0.69(0.26-1.85)		

^*^p < 0.05

Some variations were also observed in the percentages across the measures of social consequences of COVID-19. For instance, it was 7.0 and 18.3% among those with and without household food insecurity, respectively. Similarly, 12.3% of those economically reliant on partners changed their pregnancy intention while 6.2% of those economically independent changed mind about pregnancy. There were no obvious variations with respect to marital and reproductive characteristics.

Results from the univariate model identified some variables as significantly associated with changes in pregnancy intention. These include age group, wealth quintile, household food insecurity, and economic reliance on a partner. While age groups 40-44 and 45-49 years were associated with lower odds of a change in pregnancy intention, the latter variables increased the likelihood. Model I, which included all the measures of social consequences of COVID-19 restrictions, revealed that only household food insecurity (AOR = 2.79, CI: 1.29-6.04) and economic reliance on a partner (AOR = 2.04, CI: 1.01-4.12) were significantly associated with change in pregnancy intention.

From the final model fitted to identify independent predictors, age group, wealth quintile and household food insecurity were statistically significant. Women aged 25-44 were less likely than those aged 15-24 years to change their mind about pregnancy due to COVID-19 concerns Table 2, Column 5). Respondents in the lower (AOR = 0.32, CI: 0.14-0.76) and higher wealth quintile (AOR = 0.38, CI: 0.15-0.97) were less likely to change their minds about getting pregnant compared to those in the lowest quintile. Finally, respondents with experience of household food insecurity were almost three times as likely to change their minds about pregnancy (AOR = 2.72, CI: 1.23-5.99).

### Inconsistent fertility preference

**Fig. 1:**
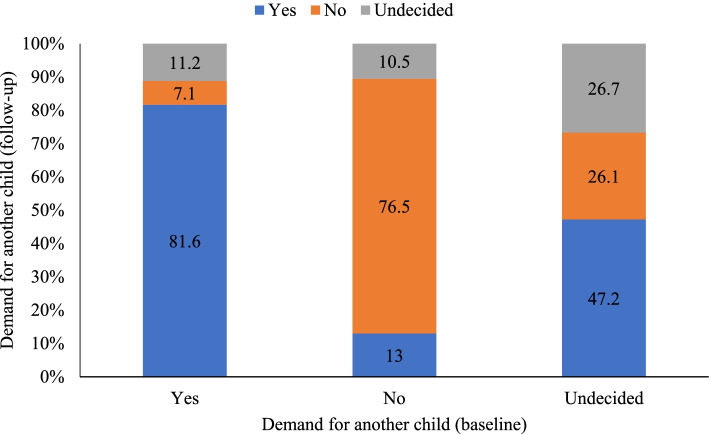
Fertility preference at Baseline and Follow-up

At baseline, 54.9% wanted another child, while 7.6% were undecided. In the follow-up survey, it was 53 and 13.1%, respectively. Figure 1 showed the changes in fertility preference between baseline and follow-up. The majority (81.6%) of those who reported wanting another child at baseline remained so at follow-up. Among the undecided at baseline, 47.2% wanted a child during follow-up, while 26.7% remained undecided.

Overall, two hundred and two (26.1%) women were found to have inconsistent fertility preferences between baseline and follow-up. Table 3 presents the distribution and factors associated with this outcome. The pattern varied across some background variables. For instance, inconsistent fertility preference was commonest in age groups 30-34 (37.2%) and 35-39 years (31.1%). It was highest among those who attained only primary education (40.3%) and lowest for tertiary (18.8%). Inconsistent fertility preference reduced as wealth quintile improved from the lowest (33.0%) to the highest (20.5%). For social consequences of COVID-19, the highest difference was observed in household food insecurity (Yes – 32.5%; No – 24.9%) and worry about future household finance (Yes – 26.7%; No – 19.5%).

**Table 3:** Frequency and factors associated with inconsistent fertility preference

Variables	Inconsistent fertility preference			
Socio-demographics	(%)	Unadjusted OR (95% CI)	Adjusted OR (95% CI)	
*Age group (Years)*			**Model I**	**Model II**
15-24	9.8	ref		ref
25-29	22.8	2.71(0.74-9.97)		2.24(0.71-7.11)
30-34	37.2	5.46(1.49-20.01)*		4.46(1.29-15.39)*
35-39	31.1	4.16(1.30-13.31)*		2.92(0.90-9.52)
40-44	21.5	2.52(0.75-8.50)		1.78(0.53-5.95)
45-49	17.4	1.94(0.58-6.48)		1.37(0.38-4.86)
*Highest education*				
None	28.9	ref		
Primary	40.3	1.66(0.76-3.62)		
Secondary	24.8	0.81(0.40-1.63)		
Tertiary	18.8	0.57(0.28-1.14)		
*Employment*				
Yes	26.5	ref		
No	24.3	0.89(0.54-1.47)		
*Place of residence*				
Urban	24.3	ref		
Rural	34.6	1.65(0.60-4.55)		
*Wealth quintile*				
Lowest	33	ref		ref
Lower	26.9	0.75(0.38-1.47)		0.79(0.41-1.54)
Middle	27.7	0.78(0.39-1.53)		0.87(0.40-1.90)
Higher	25.2	0.68(0.31-1.52)		0.78(0.32-1.91)
Highest	20.5	0.52(0.26-1.04)		0.55(0.26-1.18)
*State*				
Lagos	25.3	ref		
Kano	28.2	1.16(0.52-2.61)		
**Social consequences of COVID-19**				
*Loss of personal income*				
Small	24	ref	ref	
Moderate	31.1	1.43(0.70-2.94)	1.45(0.71-2.96)	
Large	23.5	0.97(0.55-1.73)	0.94(0.51-1.70)	
No income	24.9	1.05(0.57-1.94)	1.67(0.61-4.58)	
*Loss of household income*				
Some or partial	27.2	ref		
Total	23.7	0.83(0.56-1.24)	0.54(0.22-1.36)	
*Household food insecurity*				
No	24.9	ref	ref	ref
Yes	32.5	1.45(0.86-2.47)	1.64(1.01-2.68)*	1.47(0.86-2.51)
*Worry about future household finance*				
No	19.5	ref	ref	
Yes	26.7	1.50(0.67-3.36)	1.68(0.70-4.03)	
*Economic reliance on partner*				
No	26.4	ref	ref	
Yes	25.8	0.97(0.53-1.78)	0.99(0.54-1.83)	
**Marital and reproductive characteristics**				
*No of times married/cohabited*				
Once	25.9	ref		
More than one	28.4	1.13(0.60-2.12)		
*Family type*				
Monogamy	24.1	ref		ref
Polygynous	34	1.62(0.99-2.66)		1.42(0.85-2.36)
*Children ever-born*				
< =1	11.2	ref		ref
2	23.8	2.48(0.99-6.14)		2.25(0.87-5.84)
3	35.9	4.43(1.68-11.70)*		3.88(1.36-11.08)*
4	25.8	2.76(0.91-8.41)		2.31(0.75-7.08)
> =5	31.8	3.69(1.34-10.22)*		2.75(0.80-9.44)
*History of pregnancy termination in past 3 years*				
No	25.7	ref		
Yes	30.3	1.26(0.73-2.15)		

^*^p < 0.05

In terms of marital and reproductive characteristics, respondents in polygynous relationship (34.0%) had higher percentage of inconsistent fertility preference than those from monogamous family (24.1%). Inconsistent fertility preference was more prevalent as CEB increased. It was also higher among those with a history of pregnancy termination 3 years before the survey (Yes – 30.3%, No – 25.7%).

In the univariate model, compared to age 15-24 years, ages 30-34 years (OR = 5.46, CI: 1.49-20.01) and 35-39 years (OR = 4.16, CI: 1.30-13.31) were more likely of inconsistent fertility desire. Further, respondents with tertiary education (OR = 0.57, CI: 0.0.28-1.14) were the least likely of inconsistent fertility preference. The odds of inconsistent preference was higher among women with three (OR = 4.43, CI: 1.68-11.70) and five or more CEB (OR = 3.69, CI: 1.34-10.22).

In model I, household food insecurity (AOR = 1.64, CI: 1.01-2.68) attained a statistically significant relationship with inconsistent fertility preference. The final model showed that age group and the number of CEB were independent predictors. Those in ages 25-29 years (AOR = 4.46, CI: 1.29-15.39) were more likely of inconsistent fertility preference compared to those aged 15-24 years. In addition, the AOR for inconsistent fertility among women with 3 CEB was 3.88 (CI: 1.36-11.08) relative to one CEB.

### Disposition towards pregnancy during COVID-19

**Fig. 2:**
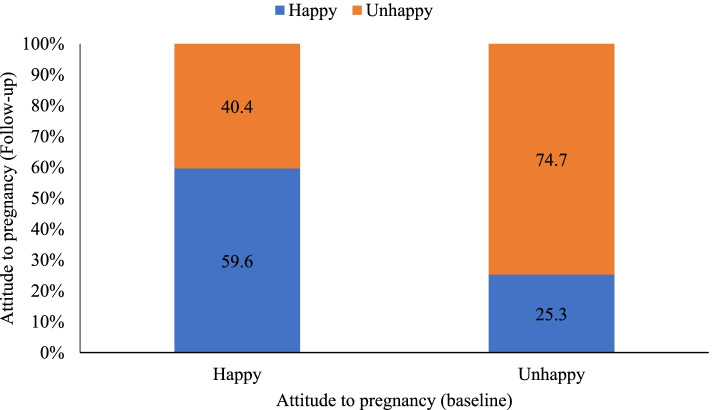
Attitude to pregnancy at Baseline and Follow-up

Overall, the percentage of women who would feel unhappy about pregnancy at baseline and follow-up was 59.4 and 60.8%, respectively. However, Fig. 2 showed that only 59.6% of those who reported that they would be happy about getting pregnant at baseline remained so during the follow-up survey. In contrast, the majority (74.7%) of those “unhappy” at baseline remained so at follow-up.

Table 4 shows the distribution and factors associated with an unhappy disposition towards pregnancy during the follow-up. The percentage unhappy ranged from 55.0% in age 15-24 years to 73.4% in the oldest age group, 45-49 years. It was lowest among those with no formal education (45.4%) and highest in those with tertiary education (66.4%). Similarly, it was higher in urban (64.2%) than rural (45.4%). The percentage with unhappy disposition towards pregnancy also ranged from 53.7% in the lowest wealth quintile to 67.6% in the highest. Respondents with a small loss of personal income (73.2%) had the highest proportion with an unhappy disposition towards pregnancy. While the percentage increased with CEB, it also varied by a history of pregnancy termination in the past 3 years before the survey (Yes – 51.8%, No – 61.7%).

**Table 4:** Frequency and factors associated with an unhappy disposition towards pregnancy amidst COVID-19

Variables	Unhappy disposition towards pregnancy during COVID-19			
Socio-demographics	(%)	Unadjusted OR (95% CI)	Adjusted OR (95% CI)	
*Age group (Years)*			**Model I**	**Model II**
15-24	55	ref		
25-29	50.7	0.84(0.44-1.63)		
30-34	57.2	1.09(0.52-2.32)		
35-39	67.6	1.71(0.79-3.73)		
40-44	62.9	1.39(0.68-2.84)		
45-49	73.4	2.26(0.94-5.46)		
*Highest education*				
None	45.4	ref		ref
Primary	62.1	1.97(1.11-3.49)*		1.93(0.89-4.18)
Secondary	61.1	1.89(1.23-2.91)*		2.08(0.99-4.36)
Tertiary	66.4	2.38(1.49-3.79)*		2.99(1.41-6.33)*
*Employment*				
Yes	60.8	ref		
No	61	1.01(0.61-1.67)		
*Place of residence*				
Urban	64.2	ref		ref
Rural	45.4	0.46(0.26-0.83)		0.57(0.25-1.30)
*Wealth quintile*				
Lowest	53.7	ref		
Lower	65.3	1.62(0.79-3.30)		
Middle	53.6	0.99(0.48-2.08)		
Higher	61.7	1.39(0.67-2.86)		
Highest	67.6	1.79(0.92-3.52)		
*State*				
Lagos	64.2	ref		
Kano	52.5	0.62(0.37-1.02)		
**Social consequences of COVID-19**				
*Loss of personal income*				
Small	73.2	ref	ref	ref
Moderate	54.6	0.44(0.22-0.87)*	0.45(0.22-0.89)*	0.39(0.18-0.86)*
Large	57.1	0.49(0.28-0.85)*	0.49(0.27-0.87)*	0.47(0.23-0.93)*
No income	64.4	0.66(0.37-1.19)	0.45(0.21-0.93)*	0.66(0.33-1.35)
*Loss of household income*				
Some or partial	58.2	ref	ref	
Total	66.5	1.42(0.96-2.11)	1.63(0.79-3.34)	
*Household food insecurity*				
No	60.1	ref	ref	
Yes	64.4	1.20(0.74-1.95)	1.15(0.67-1.95)	
*Worry about future household finance*				
No	60.9	ref	ref	
Yes	61	1.01(0.50-2.04)	0.94(0.43-2.03)	
*Economic reliance on partner*				
No	62.1	ref	ref	
Yes	59	0.88(0.60-1.29)	0.85(0.57-1.24)	
**Marital and reproductive characteristics**				
*No of times married/cohabitted*				
Once	60.8	ref		
More than one	60.9	1.00(0.50-2.03)		
*Family type*				
Monogamy	61.4	ref		
Polygynous	58.6	0.89(0.57-1.38)		
*Children ever-born*				
< =1	42.6	ref		ref
2	56.8	1.77(1.11-2.84)*		1.59(0.97-2.61)
3	67.9	2.85(1.56-5.21)*		2.95(1.58-5.53)*
4	70.7	3.25(1.74-6.06)*		3.48(1.89-6.38)*
> =5	64.6	2.46(1.29-4.69)*		4.79(2.29-10.03)*
*History of pregnancy termination in past 3 years*				
No	61.7	ref		ref
Yes	51.8	0.67(0.42-1.07)		0.70(0.42-1.19)

^*^p < 0.05

In the univariate models, education, loss of personal income and CEB were significantly related to unhappy disposition towards pregnancy (Table 4, Column 3). Women with secondary (OR = 1.89, CI = 1.23-2.910 and tertiary education (OR = 2.38, CI: 1.49-3.79) were more likely than those with no formal education to be unhappy if they got pregnant. As expected, the odds of unhappy disposition towards pregnancy increased with CEB from 1.77 (CI: 1.11-2.84) to 3.25 (CI: 1.74-6.06) in those with two and four CEB respectively.

Model I showed that loss of personal and household income was significantly associated with unhappy disposition towards pregnancy. Finally, model II showed that education, loss of personal income, and number of CEB were significantly related to unhappy disposition towards pregnancy. Women with tertiary education were 3 times as likely to be unhappy about pregnancy compared to those with no formal education (AOR = 2.99, CI: 1.41-6.33). Lastly, the likelihood of being unhappy about pregnancy increased with the number of CEB.

### Modern contraceptive use

The prevalence of modern contraceptive use (MCP) at baseline and follow-up was 30.1 and 35.1% respectively. The age pattern of MCP (Table 5), showed that it peaked at age group 35-39 years (41.0%) while it was lowest in age 45-49 years (23.2%). MCP increased from 15.5% among women with no formal education to 41.3% for tertiary education. It was also higher among urban residents (36.8%) than their rural counterparts (14.4%). Apart from the lowest wealth quintile (20.4%), the other quintiles recorded similar levels of modern contraceptive use, ranging between 33.0% (lower quintile) and 36.7% (higher quintile). An even distribution was observed in modern contraceptive use when disaggregated by the measures of social consequences of COVID-19. Women who have been married more than once had lesser prevalence of MCP (25.4%) than those married only once (33.5%). The prevalence of MCP increased with the number of CEB between 1 and 4. A substantial difference was also observed in terms of disposition to pregnancy (Happy – 19.3%, Unhappy – 41.5%).

**Table 5:** Frequency and factors associated with modern contraceptive use

Variables	Modern contraceptive use			
Socio-demographics	(%)	Unadjusted OR (95% CI)	Adjusted OR (95% CI)	
*Age group (Years)*			**Model I**	**Model II**
15-24	20.5	ref		ref
25-29	23.5	1.19(0.52-2.71)		0.58(0.27-1.23)
30-34	35.6	2.14(1.09-4.21)*		0.76(0.34-1.70)
35-39	41	2.69(1.18-6.09)*		0.71(0.28-1.82)
40-44	38.7	2.44(0.98-6.07)		0.66(0.24-1.77)
45-49	23.2	1.17(0.44-3.11)		0.24(0.09-0.69)*
*Highest education*				
None	15.5	ref		ref
Primary	29.2	2.25(0.91-5.55)		1.29(0.61-2.73)
Secondary	33.5	2.74(1.14-6.56)*		1.24(0.57-2.68)
Tertiary	41.3	3.82(1.64-8.88)*		1.64(0.73-3.68)
*Employment*				
Yes	35.1	ref		ref
No	21.4	0.50(0.26-0.96)*		0.67(0.36-1.26)
*Place of residence*				
Urban	36.8	ref		ref
Rural	14.4	0.29(0.11-0.75)*		0.56(0.17-1.79)
*Wealth quintile*				
Lowest	20.4	ref		ref
Lower	33	1.92(0.88-4.21)		1.55(0.75-3.19)
Middle	34.6	2.06(0.98-4.35)		2.04(0.94-4.40)
Higher	36.7	2.26(1.08-4.73)		2.02(0.97-4.21)
Highest	35.5	2.14(0.95-4.82)		1.49(0.59-3.73)
*State*				
Lagos	38.5	ref		ref
Kano	18.7	0.37(0.19-0.69)*		0.52(0.25-1.08)
**Social consequences of COVID-19**				
*Loss of personal income*				
Small	38.9	ref	ref	ref
Moderate	36.4	0.90(0.52-1.55)	0.91(0.53-1.57)	1.12(0.68-1.84)
Large	27.6	0.60(0.34-1.04)	0.57(0.33-.98)	0.76(0.44-1.34)
No income	32	0.74(0.42-1.30)	0.47(0.17-1.28)	0.87(0.47-1.59)
*Loss of household income*				
Some or partial	32.1	ref	ref	
Total	34.2	1.10(0.74-1.63)	1.71(0.71-4.12)	
*Household food insecurity*				
No	31.7	ref	ref	
Yes	37.9	1.31(0.83-2.06)	1.28(0.81-2.02)	
*Worry about future household finance*				
No	28.3	ref	ref	
Yes	33.1	1.25(0.71-2.22)	1.12(0.65-1.93)	
*Economic reliance on partner*				
No	35	ref	ref	
Yes	29.8	0.79(0.50-1.24)	0.79(0.51-1.22)	
**Marital and reproductive characteristics**				
*No of times married/cohabitted*				
Once	33.5	ref		
More than one	25.4	0.68(0.30-1.51)		
*Family type*				
Monogamy	34.3	ref		
Polygynous	27.1	0.71(0.41-1.23)		
*Children ever-born*				
< =1	21.4	ref		ref
2	32.5	1.77(0.98-3.20)		1.43(0.79-2.59)
3	37.6	2.22(1.30-3.79)*		1.82(1.03-3.20)*
4	42.9	2.77(1.61-4.77)*		2.45(1.36-4.39)*
> =5	29.2	1.52(0.78-2.94)		2.89(1.25-6.74)*
*History of pregnancy termination in past 3 years*				
No	33.2	ref		
Yes	27.9	0.78(0.43-1.41)		
*Changed mind*				
No	32.8	ref		
Yes	31.5	0.94(0.56-1.60)		
*Fertility preference*				
Consistent	33.6	ref		
Inconsistent	30.3	0.86(0.59-1.27)		
*Disposition to pregnancy*				
Happy	19.3	ref		ref
Unhappy	41.5	2.97(2.06-4.29)*		2.48(1.72-3.58)*

^*^p < 0.05

Results from unadjusted models is presented in Table 5, Column 3. The odds of modern contraceptive use was significantly higher among age group 30-39 compared to 15-24 years. Similarly, respondents with tertiary education were 4 times as likely as their counterparts with no formal education to use modern contraceptives. Unemployed women (OR = 0.50, CI: 0.26-0.96) and those who lived in rural areas (OR = 0.29, CI: 0.11-0.75) were less likely to use modern contraceptives. Having more than one CEB was associated with higher odds of modern contraceptive use. In addition, women with an unhappy disposition towards pregnancy were 3 times as likely to use modern contraceptives (OR = 2.97, CI: 2.06-4.29).

The final adjusted model revealed that having controlled for significant background characteristics of respondents, women aged 45-49 years (AOR = 0.24, CI: 0.09-0.69) were less likely of using modern contraceptives compared to age 15-24 years. In contrast, the odds of contraceptive use was significantly higher among those with three (AOR = 1.82, CI: 1.03-3.20), four (AOR = 2.45, CI: 1.36-4.39) and at least five CEB (AOR = 2.89, CI: 1.25-6.74) children. Unhappy disposition towards pregnancy (AOR = 2.48, CI: 1.72-3.58) was also a significant predictor of modern contraceptive use.

## Discussion

The COVID-19 pandemic and its various control measures have had enormous effects on the global economy and population health across demographic, epidemiological, and socio-economic paradigms. It has been hypothesized that in LMIC countries, childbearing intentions or fertility preferences may change because of economic and social changes brought by the pandemic. Consequently, fertility may increase or reduce depending on other factors such as access to contraception [8]. Apart from disruption of reproductive health services, which is being tackled, early evidence from SSA suggest that uptake or use of contraceptives has not suffered any downward trend [10, 11]. In this study, we add to this evolving evidence by investigating the childbearing intentions within the context of COVID-19 in Nigeria – the most populous country in SSA.

Our results showed that the COVID-19 pandemic and its control measures in Nigeria had social consequences such as total loss of household income by one-third of the study sample. Almost all were worried about future household finance. At least half of the married women had lost personal income and became economically reliant on their partners. This trend is not peculiar to Nigeria, as previous studies in HIC and other LMIC have shown similar results [15, 35]. Some of these outcomes have been shown to significantly influenced childbearing decisions in the developed countries. We explored these relationships further in Nigeria.

Our results showed that less than 10% of women changed their minds about pregnancy due to COVID-19 concerns, and this was found to be more prevalent among women in the higher wealth quintile and those whose households had experienced some forms of food insecurity. It was also associated with economic reliance on partner. The proportion of women who changed their pregnancy intention is far less than what has been reported in some developed countries, where at least one-third opted to postpone pregnancy/childbearing due to COVID-19 and its attendant economic hardship [16–18]. The difference in the percentage of women who changed minds about pregnancy is not surprising because the contexts differ in various ways. For instance, the two settings are in different stages of demographic transition, with Nigeria being in the high fertility phase. Besides, fertility aspirations are higher in Nigeria than in many developed countries. The difference in change of pregnancy intentions may also have reflected the COVID-19 contexts. Obviously, the pandemic has had a greater impact in terms of mortality and morbidity in developed countries than in SSA and Nigeria in particular [8]. Historical evidence suggests that in the short term, after a pandemic or natural disaster or any other population shock, birth rates are usually lower but may pick up later [8]. The fact that household food security and economic reliance on partner was significantly related to change in pregnancy intention among Nigerian women shows the devastating effects of the pandemic on socio-economic status. This finding is consistent with prior evidence in another SSA country [36]. Perhaps, fears that pregnancy will bring additional children and extra mouth to be fed makes people change their minds. This is expected in a setting where poverty is ravaging.

Furthermore, our results showed that about one-quarter of study participants had inconsistent fertility preferences within a short space of 6 months. This was common in age 25-39 years, and the odds increased with the number of CEB and household food insecurity. The literature on fertility preferences in SSA suggests that childbearing desire is rarely constant but changes with people’s circumstances [14, 37, 38]. The pattern of changes showed that women with “undecided” fertility preference at baseline constituted a large portion of those found to have inconsistent fertility preference. The fact that the odds of inconsistent fertility preference increased with CEB suggests that the women had challenges in setting fertility goals and sticking to it. Though, the idea of fertility planning is not popular in Nigeria. This may explain why high parity women could have inconsistent preferences. Reproductive health education would be necessary to enlighten women about fertility goals/planning and working to actualise it irrespective of pandemic or any other public health challenge. Childbearing intentions should not be circumstantial but a carefully thought-out decision with adequate preparation.

Our result further revealed that two-thirds reported that they would be unhappy if they got pregnant. The likelihood increased with CEB, wealth quintile and loss of income. Surprisingly, there was no difference between this proportion at baseline and follow-up. Therefore, this emotional response may not be due to the pandemic. Curiously, 54% of the women want another child. These results revealed a high fertility preference with a desire to delay childbearing irrespective of the COVID-19 pandemic. The critical question is how well does the inherent desire to delay pregnancy translate into contraceptive uptake?

Further exploration of the data showed that women with unhappy disposition to pregnancy during the pandemic lockdown were more likely to use modern contraceptives. This is a window of opportunity that can be exploited as part of family planning advocacy. The message focus could be, “if you get pregnant now, will you be happy? If the answer is no, then go for family planning”. In short, it is necessary to tweak the motivations for family planning use among Nigerian women [39, 40]. Multiple channels of intervention would be necessary to achieve results in this regard. None of the social consequences of COVID-19 was a significant predictor of contraceptive use. This is in tandem with recent studies which found that the pandemic has not adversely affected the need and use of contraceptives [11, 41].

The prevalence of modern contraceptive use found in the two States deserve some comments. The observed level should not be taken as reflective of modern Contraceptive Prevalence Rate (mCPR) in Nigeria as a whole because Lagos and Kano falls in two extremes of the indicator. The result for Lagos fell within the rates reported for previous surveys while Kano was slightly higher [25]. In fact, previous study based on 2017, 2018 and 2019 rounds of PMA data from Lagos showed that contraceptive has been persistently high [11]. Unfortunately, data for Kano was not included in the previous analysis. A slightly higher mCPR for Kano may be due to two reasons. First, it could be that truly, there has been an improvement in mCPR among women in the State because there are a number of intervention programmes targeted at reproductive health, maternal and child health in Nigeria [42]. Secondly, we observed that there was a substantial dropout between baseline and follow-up in Kano survey due to non-ownership of phones and absence of consent for repeat interview. Although the data was reweighted to correct for this, it is still possible that the women who consented to follow-up were more educated, had higher education and socio-economic status. Subsequently, these may report higher level of mCPR than obtained in the general population of women in Kano. This suspicion would need indepth systematic assessment for clearer understanding of the issues.

The limitations of this study should be kept in view. First, data were collected at the peak of COVID-19 lockdown restrictions in Nigeria. Therefore, the results only relate to the short-term following the pandemic. Although lockdown has since been lifted and economic activities have gradually picked up, successive waves of the pandemic has been heralded. Similarly, vaccination has ommenced, although with a very slow rollout. The medium and long-term outlook for childbearing intentions, fertility levels and reproductive health, in general, cannot be concluded based on this data. However, we do not expect the social consequences of COVID-19 on childbearing intentions to change. This is because the key issues are mostly economical and less of COVID-19. Economic instability does not seem to have had much impact on fertility regimes in Nigeria; otherwise, CEB should not have been higher among the poor.

Secondly, the data analysed were collected via telephone interviews. One study suggests that responses in phone interviews do differ from face-face [43]. This should not affect the general pattern of the results and the inferences drawn from them. The fact that not all women who participated in the baseline data collection were part of the follow-up should not introduce selection bias for our analytical sample. The third obvious limitation is the lack of data on sexual behaviour. We could not assess if the COVID-19 pandemic occasioned changes in sexual behaviour and whether it affected childbearing intentions and modern contraceptive use. Despite these limitations, this study has made a modest contribution to the needed empirical evidence on the implications of COVID-19 lockdown on reproductive behaviour among Nigerian women.

## Conclusion

In this study, we documented some social consequences of COVID-19 among childbearing women in Nigeria. These include loss of personal income, partial or total loss of household income, greater economic reliance on partner, and household food insecurity. Total loss of household income and food insecurity were associated with unstable fertility intentions. About two-thirds of women reported that they would be unhappy if pregnant at the survey time. Such women were more likely to use modern contraceptive methods while none of the social consequences of COVID-19 was associated with the former. That is, indirectly, the pandemic lockdown is a catalyst for contraceptive uptake. Background characteristics such as age, wealth quintile, education and parity retained their usual relationships with fertility intentions and contraceptive use.

These findings provide some glitters of hope for improvement and sustenance of family planning, sexual and reproductive health programmes. For instance, the relationship between the social consequences of COVID-19, fertility intentions and modern contraceptive use have two implications. First, married women and their spouses need to be enlightened about fertility planning such that their childbearing intentions would not be tied to economic and other social circumstances. Secondly, since COVID-19 lockdown does not directly affect contraceptive use, it means that family planning (FP) programmes should be sustained so that the progress already achieved can continue to be improved upon. At least, results are showing that there may be no fears about a reversal in the progress of FP uptake because of the COVID-19 pandemic. The finding about the emotional response to pregnancy and its association with contraceptive use provide a clue on another strategy for family planning advocacy. Stakeholders in charge of FP demand creation and communication can take a further in-depth look at this aspect. Messages can be designed to help women assess their emotional disposition to pregnancy and make an informed decision about contraceptive use. Future large-scale studies with a broader scope encompassing other components of sexual and reproductive health would be necessary for continuous monitoring of the medium and long-term consequences of the pandemic.

## Data Availability

The datasets analysed for the current study are available in the PMA online repository [26].

## Abbreviations

CEB: Children-Ever-Born
CPR: Contraceptive Prevalence Rate
LMIC: Low-Middle Income Countries
MCH: Maternal and Child Health
OR: Odds Ratio
PMA: Performance Monitoring for Action
SDG: Sustainable Development Goal
SRH: Sexual and Reproductive Health
SSA: Sub-Saharan Africa
